# Supplementary material for: Deficiency of mannose-binding lectin is a risk of Pneumocystis jirovecii pneumonia in a natural history cohort of people living with HIV/AIDS in Northern Thailand
Source: PLoS One. 2020 Dec 23;15(12):e0242438. doi: 10.1371/journal.pone.0242438 (PMC7757797; doi:10.1371/journal.pone.0242438)
Supplement: S1 Fig — The solid line means “low”, and dashed line means intermediate and high combined. The difference between “low “vs “int + high” were analyzed by log-rank test; however, there were no significance (p = 0.44). (DOCX) [file pone.0242438.s001.docx]

**S1 Fig. Cumulative probability curves of PCP during follow-up periods (CD4 count <50, n=171)**Solid line: low，Dashed: Int + High


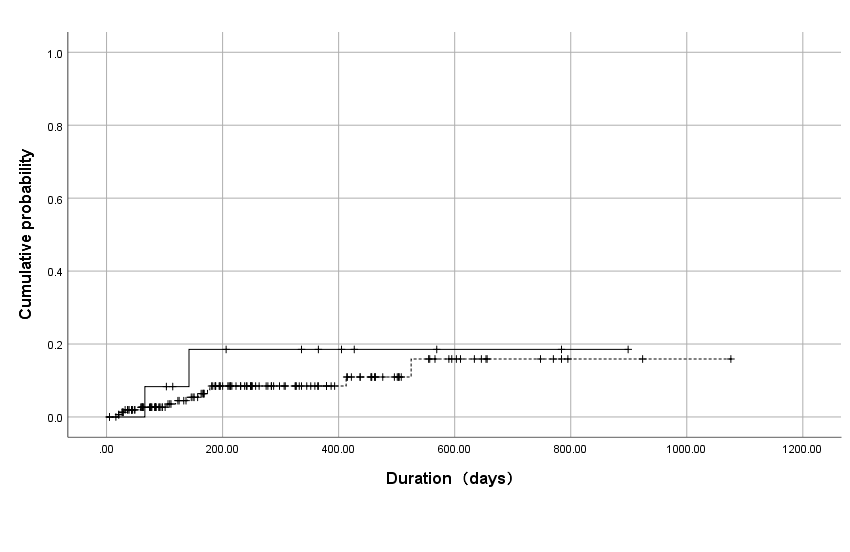


*p*=0.44
