# Supplementary figures and images for: Deficiency of mannose-binding lectin is a risk of Pneumocystis jirovecii pneumonia in a natural history cohort of people living with HIV/AIDS in Northern Thailand
Source: PLoS One. 2020 Dec 23;15(12):e0242438. doi: 10.1371/journal.pone.0242438 (PMC7757797; doi:10.1371/journal.pone.0242438)

**S2 Fig.** **Box plots of plasma MBL concentrations with or without PCP during the follow-up period (n=231)**


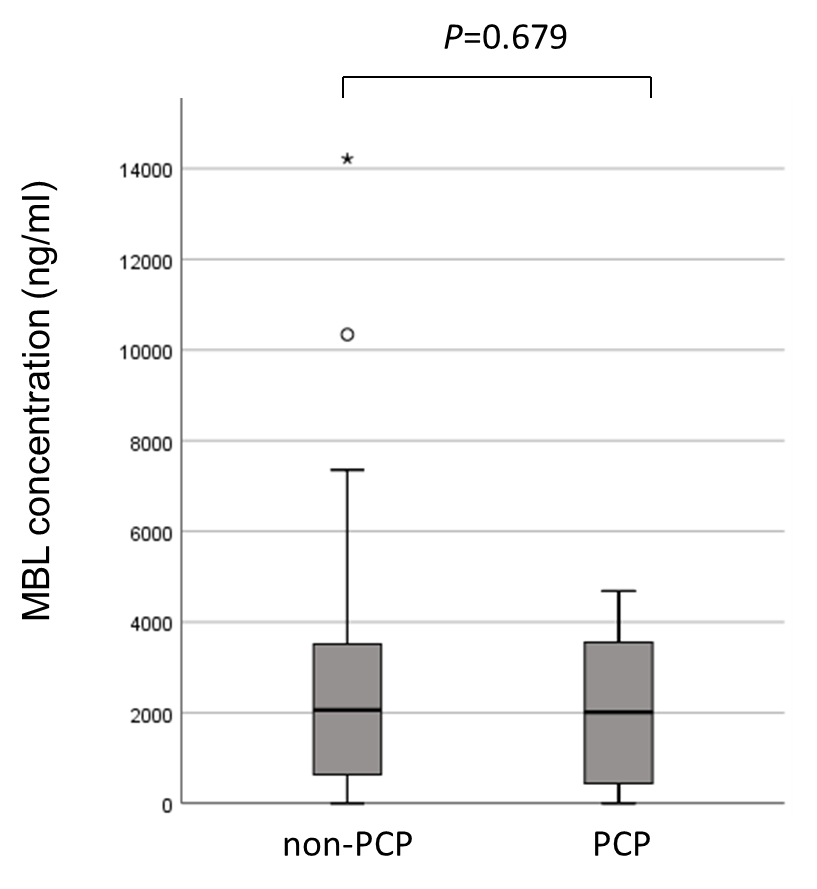

Supplement: S2 Fig — MBL concentrations were plotted in two group of patients if they have a new episode of PCP (n = 19, plasma MBL was 0.0–4683.4/ng/ml, median 2008.9 ng/ml) or not (n = 212, 0.0–14213.3, median 2057.8 ng/ml) during the follow-up period. The median of each two group was tested by Mann-Whilney U test; however, there were no significance (p = 0.679). (DOCX) [file pone.0242438.s002.docx]
