# Supplementary material for: Deficiency of mannose-binding lectin is a risk of Pneumocystis jirovecii pneumonia in a natural history cohort of people living with HIV/AIDS in Northern Thailand
Source: PLoS One. 2020 Dec 23;15(12):e0242438. doi: 10.1371/journal.pone.0242438 (PMC7757797; doi:10.1371/journal.pone.0242438)
Supplement: Attachment — Submitted filename: PLOSE ONE Review.docx [file pone.0242438.s004.docx]

The manuscript by Yanagisawa and colleagues describes the association between mannose-binding lectin and susceptibility to Pneumocystis jiroveccii pneumonia (PCP) in HIV+ ART-naive patients in Northern Thailand. PCP was diagnosed in 15% of patients and the authors report MBL expression to be associated with PCP onset in patients with CD4 counts > 50 /ul.

The study is straightforward, and the data are well presented. However, the Kaplan Meir curves showing decreased likelihood of PCP over time is confusing. Also, the lower risk of PCP incidence in patients with CD4 counts < 50/ul is unclear. Were other important confounders not accounted for such as susceptibility to other OIs? Another caveat is that presence of coinfections impacting liver function could impact MBL levels and this confounder was not controlled for.
