# Supplementary material for: Deficiency of mannose-binding lectin is a risk of Pneumocystis jirovecii pneumonia in a natural history cohort of people living with HIV/AIDS in Northern Thailand
Source: PLoS One. 2020 Dec 23;15(12):e0242438. doi: 10.1371/journal.pone.0242438 (PMC7757797; doi:10.1371/journal.pone.0242438)
Supplement: Attachment — Submitted filename: Response to Reviewers_201030.docx [file pone.0242438.s005.docx]

Response to the reviewers

**To reviewer #1:**

“The manuscript by Yanagisawa and colleagues describes the association between mannose-binding lectin and susceptibility to *Pneumocystis jiroveccii* pneumonia (PCP) in HIV+ ART-naive patients in Northern Thailand. PCP was diagnosed in 15% of patients and the authors report MBL expression to be associated with PCP onset in patients with CD4 counts > 50 /ul. The study is straightforward, and the data are well presented. However, the Kaplan Meir curves showing decreased likelihood of PCP over time is confusing. “

**Thank you for your suggestion. The Y-axis of Kaplan-Meier curves in Fig1 in the first manuscript may give misunderstanding that the incidence of the PCP looks to be decreased over time as you mentioned. In the revised manuscript, we modified Fig1. that Y-axis show the increase of PCP incidence as follows.**

**Revised Fig 1.**

a. Total, n=569, log-rank *p*=0.011

“Also, the lower risk of PCP incidence in patients with CD4 counts < 50/ul is unclear. ”

**Thank you for your suggestion. In this study, all patients having baseline CD4<200/µl were taken TMP/SMX for the PCP prophylaxis, so that the incidence of PCP in patients with CD4<50/µl group was modified by this medication. However, after the re-categorization of MBL expression type in the revised manuscript (deficient/low/intermediate/high), the cut-off level of CD4 counts below or over 50/µl were not a critical factor of PCP. Therefore, we added Fig 1. b (CD4<200) and c (≧200) in revised version to clarify the impact of PCP prophylaxis that they have been taken or not. As a result, we confirmed that deficient MBL expression type was an independent risk factor of PCP incidence whether CD4 counts were below nor over 200/µl through K-M analysis, univariate and multivariate Cox’s proportional hazard analysis (P10, line 204-210; P11, line 220-226).**

b. CD4 count <200, n=293, log-rank *p*=0.030

c. CD4 count ≧200, n=274, log-rank *p*=0.021

“Were other important confounders not accounted for such as susceptibility to other OIs? Another caveat is that presence of coinfections impacting liver function could impact MBL levels and this confounder was not controlled for.”

**Thanks for your suggestion. Because the data associated with liver function (i.e. liver transaminase, prothrombin time and choline-esterase activity) and all of plasma samples were not available, we could not conduct the analysis including these confounders. We also agree that MBL synthesis can be affected by many other confounders including liver functions, co-infections such as hepatitis B and C virus and other OIs. In consideration of these concerns, we retried cross-sectional, univariate and multivariate analysis including HBsAg and HCV-Ab as additional confounding variables. As a result, deficient expression type remained** **as a significant risk factor of PCP histories (P9, line 190-193, Table 2), and its incidence (P11, line 220-226, Table 3). We believe these data show the critical role of MBL expression type at the onset of PCP upon the co-infection of hepatitis B and C virus.**

**To reviewer #2:**

The only suggestion that I might offer involves the distinction between MBL low genotypes and MBL deficiency.

**As you mentioned, patients classified “low” in our first manuscript includes complete deficiency to relatively low expression. Therefore, their plasma MBL concentrations distributed between undetectable to relatively low levels (shown in Fig 2). We re-analyzed our data according to your kind advice that we should distinguish the deficiency and low. As a result, we achieved more impressive results. Briefly, we confirmed that MBL deficiency associated with PCP histories (Table 2), cumulative provability (Fig 1) and incidence independently upon sex, age, CD4 and log_10_VL (Table 3). We appreciate your great advice and believe that our revised data improved to show the association between MBL expression type and PCP onset more clearly through the new manuscript.**

Although the authors demonstrate a very strong correlation between genotype and deficiency, their analysis of susceptibility to PCP is with the genotype, not the phenotype. Perhaps they could make this distinction in their concluding remarks.

**As you mentioned, we agree the limitation that we could not analyze the MBL phenotype and the PCP onset, because all of plasma samples were not available. Therefore, we added this discussion that further studies are needed to clarify the association of MBL phenotype and PCP onset (P15, line 314-318).**

**Response to Journal Requirements:**

When submitting your revision, we need you to address these additional requirements.

1. Please ensure that your manuscript meets PLOS ONE's style requirements, including those for file naming.

**We apologize that first manuscript was not suitable for the PLOS ONE format. We have made the revised manuscript according to the instruction included in the URL you provided, then please confirm it again.**

2. Please provide the following details about the stored biological samples used in your study:

(1) source of the samples　(2) whether the samples were completely de-identified before researchers accessed the samples. We note that you state that "stored samples derived from the Lampang HIV cohort in “unlinked anonymous” pattern can be used in subsequent analyses without requiring additional ethical approval." Please clarify whether the samples were "unlinked anonymous" before you accessed them. Please add this information to the methods section and your ethics statement in your online submission form.

**Thanks for your suggestion. (1) We used the genomic DNA extracted by the standard protocol of phenol-chloroform method from the peripheral blood of registered patients. We clarified this preparation steps in the revised manuscript (P6, line 128-129). (2) This study was performed by the second use of anonymity samples and information provided in Lampang HIV cohort. Before the analysis and manuscript preparation, authors have not been provided any information that can identify the individuals registered in cohort. We added this point in ethical statement (P8, line 177-179), and input in the submission form.**

3. PLOS requires an ORCID iD for the corresponding author in Editorial Manager on papers submitted after December 6th, 2016. Please ensure that you have an ORCID iD and that it is validated in Editorial Manager. To do this, go to ‘Update my Information’ (in the upper left-hand corner of the main menu), and click on the Fetch/Validate link next to the ORCID field. This will take you to the ORCID site and allow you to create a new iD or authenticate a pre-existing iD in Editorial Manager. Please see the following video for instructions on linking an ORCID iD to your Editorial Manager account:

https://www.youtube.com/watch?v=_xcclfuvtxQ

**I (Kunio Yanagisawa) have already applied my information to ORCID form that you indicated.**

4. We note that you have included the phrase “data not shown” in your manuscript. Unfortunately, this does not meet our data sharing requirements. PLOS does not permit references to inaccessible data. We require that authors provide all relevant data within the paper, Supporting Information files, or in an acceptable, public repository. Please add a citation to support this phrase or upload the data that corresponds with these findings to a stable repository (such as Figshare or Dryad) and provide and URLs, DOIs, or accession numbers that may be used to access these data. Or, if the data are not a core part of the research being presented in your study, we ask that you remove the phrase that refers to these data.

**We apologize our lack of understanding that the description of "data not shown " is not accepted. We deleted this description, then the data necessary to make discussion was included in supportive information at the end of revised manuscript.**
